# Emotion regulation and cognitive function as mediating factors for the association between lifetime abuse and risky behaviors in women of color

**DOI:** 10.1371/journal.pone.0279325

**Published:** 2023-10-30

**Authors:** Karina Villalba, Lisa H. Domenico, Robert L. Cook, Julia O’Connor, Kyndester Michael-Samaroo, Maria Jose Del Pino Espejo, Pilar Martin, Jessy G. Dévieux

**Affiliations:** 1 College of Medicine, University of Central Florida, Orlando, Florida, United States of America; 2 College of Nursing, University of Florida, Gainesville, Florida, United States of America; 3 Department of Epidemiology, University of Florida, Gainesville, Florida, United States of America; 4 School of Social Work, University of Central Florida, Orlando, Florida, United States of America; 5 Department of Sociology, Universidad Pablo De Olavide, Seville, Spain; 6 College of Nursing and Public Health, Adelphi University, Garden City, New York, United States of America; 7 Department of Health Promotion and Disease Prevention, Florida International University, Miami, Florida, United States of America; The Hong Kong Polytechnic University, HONG KONG

## Abstract

**Background:**

The relationship between lifetime abuse (i.e., childhood abuse, intimate partner violence) and risky behaviors is well established. One proposed mechanism is poor emotion regulation and executive functioning, as a potential mechanism that may explain the relationship between lifetime abuse and risky behaviors. However, research on executive functioning and emotion regulation as mediators of this relationship has been limited. In the present study, we examined this association. We hypothesized that lifetime abuse would be significantly associated with executive function and emotion regulation which in turn would be associated with greater alcohol use and risky sex.

**Methods:**

This cross-sectional study included 150 women with a history of lifetime abuse who were assessed for hazardous alcohol use using the AUDIT Score; emotion regulation was measured using the Difficulties with Emotion Regulation Scale (DERS); risky sex was measured using the question: "in the last 90 days, how many people did you have anal or vaginal sex without using a condom? Executive function was assessed using the NIH Toolbox.

**Results:**

The mediation model followed the self-regulation theory, which proposes executive function as the higher-order cognitive process. Results showed that executive function deficit and poor emotion regulation significantly mediated the relationship between lifetime abuse and hazardous alcohol use (indirect effect = .097, SE .031, 95% CI = .035 to .158).

**Conclusion:**

Our findings suggest a higher-order cognitive process with executive function promoting emotion regulation as a potential mechanism for alcohol problems in women of color who experienced lifetime abuse.

## 1. Introduction

National data indicate that one in four women have experienced violence or abuse (e.g., psychological, physical, sexual) in their lifetime [1], with findings from lifespan studies supporting cumulative abuse as a dose–response exposure for different types of abuse across a lifetime [2]. African American and Hispanic/Latina women have higher rates of childhood abuse and intimate partner violence compared to other racial and ethnic groups in the United States [3]. According to the Centers for Disease Control and Prevention (CDC), the lifetime prevalence of intimate partner violence (IPV) is 41.2% for non-Hispanic Black women and 35.5% for Hispanic women compared to 31.5% for non-Hispanic White women [4]. Similarly, childhood abuse is 24.8% for Hispanics, 23.9% for Blacks compared to 15.3% non-Hispanic White [1]. One of the long-term adverse implications of childhood abuse is revictimization in adulthood, with higher rates among women who experienced childhood abuse than among women who did not experience childhood abuse [5, 6]. We conceptualized lifetime abuse as the cumulative effect of exposure across a woman’s lifespan (e.g., childhood, adolescence, adulthood) from one or various perpetrators in one or several settings (e.g., home, school, work) involving at least one type of violence (psychological, physical, and sexual abuse [2, 7–9].

Lifetime abuse is associated with multiple adverse outcomes, including alcohol use disorders (AUD) and risky sex. Women who experience lifetime abuse are more likely to report high-risk partners (e.g., intravenous drug users, HIV-positive individuals). They tend to engage in risky sexual behaviors, including inconsistent condom use, promiscuity, and early sexual initiation [10–12]. There is also growing evidence suggesting that women who experienced lifetime abuse can be less sexually assertive. This behavior can result in less condom insistence and a greater likelihood of unprotected sex [13]. Substantial evidence shows that women who experienced lifetime abuse are twice as likely to suffer from AUD when compared to women who experienced either childhood abuse or intimate partner violence alone [14–16]. These women are more likely to begin drinking at a younger age and report more problems related to alcohol use [17–19].

Women who experienced lifetime abuse have long-term difficulties with emotion regulation strategies and engage in emotionally avoidant coping behaviors [20–24]. In addition, women in violent relationships are usually subjected to emotional invalidation by their partners, which in turn can lead to developing more frequent and long-lasting periods of inability to control their emotions and use alcohol as a negative coping behavior [25, 26]. Women who experience lifetime abuse are also at a higher risk of developing cognitive problems, including executive dysfunction. Several theories have been proposed to explain the relationship between lifetime abuse and risky behaviors. The Self-regulation Model proposes a causal order that includes executive function as a higher-order cognitive process that contributes to emotion regulation [27]. Self-regulation failure is associated with negative emotional states, resulting in executive function deficit and poor emotional regulation, ultimately leading to maladaptive coping behaviors [28]. Executive function is a higher-order cognitive process that includes planning, cognitive flexibility, social cognition, emotion regulation, and behavioral competencies such as attitudes and behaviors [28]. Emotion regulation can be intrinsic or extrinsic; intrinsic refers to one regulating their own emotions (i.e., self-regulatory processes); extrinsic refers to an individual regulating someone else’s emotions (e.g., a parent regulating the emotions of an infant by soothing her [29]. Developmental psychology studies have shown that one of the roles of a parent is to teach a child emotion regulation strategies; however, children growing up in abusive environments may not be able to develop an intrinsic regulation mechanism because of the lack of support from their parents [30, 31].

While there is evidence demonstrating a relationship between violence or abuse, alcohol use and poor emotion regulation there is relatively limited research exploring the indirect effect of executive functioning and emotion regulation through lifetime abuse and risky behaviors [16, 27, 32]. The present study aimed to provide additional evidence for the association between cognitive function, and risky behaviors. First, we conceptualized lifetime abuse as emotional, physical, or sexual revictimization across two periods (childhood and adulthood). This is important as much of the prior research has focused on a single type of abuse and/or time period. Second, we hypothesized that poor emotion regulation and deficit in cognitive function will be indirectly associated with lifetime abuse and risk behaviors. Previous research shows that women who experienced abuse may be at increased risk for relying on maladaptive strategies for self-regulating negative emotions or coping with negative experiences. As such, we expect that lifetime abuse would be significantly associated with difficulty in emotion regulation. Therefore, we propose to evaluate executive function and emotion regulation as mediating pathway in the relationship between lifetime abuse and risk behavioral outcomes. We hypothesized that deficit in executive function promote poor emotion regulation and mediate the association between lifetime abuse, hazardous alcohol use, and risky sex.

## 2. Methods

### 2.1. Participants and procedure

This was a cross-sectional study with 150 women of color (African American, Hispanic/Latina) reporting a history of childhood abuse and intimate partner violence between 2018 and 2019. The sample consisted of women of color (Hispanic and African American) at risk for HIV recruited from four community-based organizations, including Care 4U, Empower U, and Care Resources, with two locations, in a densely populated, multicultural, urban area in South Florida. The recruitment was in-person, using flyers and presentations. To be eligible, participants had to be between 18 and 50 years old; have a history of childhood abuse and/or intimate partner violence; have at least one episode of a sexual encounter in the last three months; a history of alcohol use in the last three months; fluent in spoken English and/or Spanish; and be able to provide informed consent. This study was approved by the Institutional Review Board of Florida International University, and all women provided written informed consent. We followed a strict protocol for privacy and confidentiality.

### 2.2 Measures

The survey was available in both English and Spanish. The survey instruments that were not validated in Spanish were translated into Spanish and then back-translated and reviewed by a Cultural Linguistic Group to ensure cultural appropriateness and relevance. The survey instrument measured the following domains used within the current analysis.

#### 2.2.1 Demographics

Self-reported sociodemographic information included age, race and ethnicity, employment status, and education.

### 2.3 Predictors

#### 2.3.1 Intimate partner violence

The World Health Organization (WHO) Self-reported Questionnaire [33] measured intimate partner violence. This is a culturally adapted questionnaire from a multi-country study conducted by the WHO. The 13-item questionnaire asked participants about the type of abuse (emotional, physical, sexual) and the timing of the abuse (last 12 months, lifetime). Responses to these questions were ’Yes (= 1)’ and ’No (= 0)’. All questions were added to compute a composite score that ranged from 0 to 13 for IPV, with higher scores indicating higher abuse.

#### 2.3.2 Childhood abuse

The Childhood Trauma Questionnaire is a 28-item validated instrument used to retrospectively assess emotional abuse, emotional neglect, physical abuse, physical neglect and sexual abuse [34]. Participants were asked about their experiences with types of abuse during their childhood. All questions utilized a five-point Likert scale with responses ranging from "never true" to "very often," with a total of four subscales each with 4–5 questions measuring each type of abuse with a maximum possible score of 25, with higher scores reflecting higher childhood abuse. For this study we only used emotional, physical and sexual abuse.

#### 2.3.3 Lifetime abuse

For this study, we developed a continuous additive weighted composite score that included variables related to emotional, physical, and sexual childhood abuse and emotional, physical, and sexual, intimate partner violence.

### 2.4 Mediators

#### 2.4.1 Emotion regulation

This construct was measured using the Difficulties with Emotion Regulation (DERS) scale [35], which evaluates emotion regulation difficulties in six areas, including nonacceptance of emotional responses, difficulties engaging in goal-directed behaviors, impulse-control difficulties, lack of emotional awareness, limited access to emotion regulation strategies, and lack of emotional clarity. Scores ranged between 36 to 180, with prior research suggesting the clinical range on DERS total scores between 80–127. Higher scores indicate greater difficulties with emotion regulation.

***Executive function*** was measured using the NIH Toolbox in English and Spanish using normative data from English- and Spanish-speaking populations [36]. Executive control was measured using the Flanker Inhibitory Control and Attention and the Dimensional Change Card Sort test. We pooled the Flanker Test, which measures the allocation of an individual’s limited capacities to deal with environmental stimulation, and the Dimensional Change Card Sort Test, which measures the capacity to plan, organize and monitor the executive of behaviors that are strategically directed, in a goal-oriented manner as predictors of executive functioning. Higher scores indicated higher levels of cognitive functioning. A standard score at or near 100 indicates cognitive ability that is average compared with others nationally. Standard scores around 115 suggest above-average cognitive ability, while scores around 130 suggest superior ability (in the top 2 percent nationally, based on Toolbox normative data). Conversely, a standard score of around 85 suggests below-average cognitive ability and a score in the range of 70 or below (bottom 2 percent) suggests very low cognitive functioning.

### 2.5 Outcomes

#### 2.5.1 Risky sex

We included: To measure sexual risk, we used a continuous variable using the following question: "*in the last 90 days*, *how many people did you have anal or vaginal sex with without using a condom*?" This is a self-report measure.

#### 2.5.2 Alcohol use

The Alcohol Use Disorders Identification Test (AUDIT) is a 10-item survey that measures alcohol consumption, dependence symptoms, and personal and social harm reflective of drinking over the past 30 days. It covers the areas of alcohol consumption, drinking behavior, and alcohol-related problems. Responses are scored from 0 to 4, with a maximum possible score of 40, with higher scores indicating problematic drinking. A score of 8 or more is associated with harmful or hazardous drinking. For this study, hazardous alcohol use was a continuous variable defined as hazardous alcohol use with a score ≥ 8.

## 3. Data analysis

The data analytic plan included three steps. First, descriptive statistics were computed; second, correlations were estimated for all variables in the mediation models; and third, to measure hazardous alcohol use and risky sex mediating models, we measured the direct and indirect associations between lifetime abuse, executive function, emotion regulation, hazardous alcohol use, and risky sex.

Descriptive data were analyzed using SPSS 26.0 (IMB, Chicago, IL) and were checked for normality, outliers, and missing values. Descriptive statistics (mean, standard deviations, skewness, kurtosis) were calculated to describe sample characteristics and tested for normality assumptions for all continuous variables. Correlation analyses were conducted for all continuous variables. Bivariate analysis of demographic and key variables was conducted utilizing chi-square tests. Demographic and other continuous variables significantly associated at the bivariate level with alcohol use/risky sex were included in subsequent models as covariates.

We performed two serial mediation models–Model 1, hazardous alcohol use, and Model 2, risky sex. These models used the serial mediation model to predict the relationship between executive function deficit and poor emotion regulation as mediating variables in the pathway between lifetime abuse and risk behaviors. The direct and indirect effects of lifetime abuse on hazardous alcohol use and risky sex via the mediating mechanism of executive function and emotion regulation were assessed with multiple ordinary least squares (OLS) regressions through the SPSS macro PROCESS version 3.203 [37]. This method simultaneously estimates the direct association of X on Y (c’-path), the direct association of X on M (a-path), the direct association of M on Y (b-path), and the indirect association of X (lifetime abuse) on Y (hazardous alcohol use or risky sex) via M (poor emotion regulation and executive function deficit). The indirect effect (i.e., mediation) was tested using 10,000 resampling bias-corrected bootstrap confidence intervals (95% CI). Indirect effect models were run separately for hazardous alcohol use and risky sex. Lifetime abuse was entered as the X (independent) variable. Executive function deficit and poor emotion regulation were entered as serial mediating variables into each model [hazardous alcohol use and risky sex (i.e., number of partners in the last 90 days)]. Hazardous alcohol use and risky sex were entered into the models individually as the Y (dependent) variable. Age and level of education were entered into all models as control variables. We chose the OLS regression as a preferred method as it offers the least Type I and Type II errors [37], and it has greater power to detect mediational effects than similar approaches [38].

## 4. Results

### 4.1 Descriptive analysis

The mean age was 42 (SD = 11.0) years old (see Table 1). Forty-seven percent were Black, with 54% of the sample self-identified as Hispanic. The mean alcohol use among the women was 10.4 (SD = 8.6), indicating hazardous alcohol use. The mean number of sexual partners without using a condom in the last 90 days was 2 (SD = .97). The mean score for emotion regulation was 84.2 (SD = 24.5), with higher scores indicating difficulties in emotion regulation; similarly, the executive function score of 72 (SD = 12.5) indicated lower executive functioning compared to the normative data which may indicate difficulties in general functioning.

**Table 1:** Sociodemographic characteristics among women of color with lifetime abuse.

Demographic Characteristics	n = 150
**Age, mean (SD)**	42 (11)
**Race, No (%)**	
White	47 (31)
Black	72 (47)
Other	32 (22)
**Hispanic, No (%)**	83 (54)
**Marital Status, No (%)**	
Single	62 (41)
Married	37 (24)
Separated/living with a partner	45 (35)
**Education, No (%)**	
Some high school or lower	43 (28)
High school diploma	48 (31)
Some College	29 (19)
College graduate	32 (22)
**Income No (%)**	
$5,000 or less	41 (27)
$5,000 - $10,000	51 (34)
$10,000 - $19,999	38 (25)
$30,000 or more	21(14)
**Risky Behaviors No (%)**	
Alcohol use severity score mean (SD)	10.4 (8.6)
Risky sex (# of sexual partners without a condom in the last 90 days)	2 (.97)
**Regulation, mean (SD)**	
Emotion regulation	84.2 (24.5)
Executive function	72 (12.5)

### 4.2 Correlational analysis

We found a positive correlation between lifetime abuse and poor emotion regulation (.284, *p* < .001). Risky sex was positively correlated with poor emotion regulation (.324, *p* < .001) and hazardous alcohol use (.183 *p* < .05; See Table 2).

**Table 2:** Study variables correlation analysis.

	**1**	**2**	**3**	**4**	**5**	**6**
**(1) Age**	.					
**(2) Risky Sex**	-.196*	.				
**(3) Lifetime abuse**	-.068	.144	**.**			
**(4) Poor emotion regulation**	.069	.324**	.284**	.		
**(5) Executive function deficit**	.116	-.101	**.**138	-.188	**.**	
**(6) Alcohol use severity**	.183*	.250**	.129	.505**	.325**	**.**

### 4.3 Mediation results

We tested two serial mediation models with differing outcome variables. Model 1 –hazardous alcohol use; Model 2 –risky sex. The mediators were ordered between the predictor and the outcome. The order of the mediators followed the self-regulation theory; we first included executive function as the higher-order cognitive process, followed by emotion regulation (27). Since this is a cross-sectional study, we cannot imply causality, but results may pave the way for future longitudinal mediation analysis measuring key study constructs (see Table 3).

**Table 3:** Direct and indirect analysis for alcohol use severity and risky sex among women of color with a history of lifetime abuse.

Alcohol Use Model 1				Bootstrapping 95% CI	
	Effect	SE	Boot SE	Lower 2.5%	Upper 2.5%
**Direct Effect**					
Lifetime abuse → executive function deficit	**.15**	**.05**		**.036**	**.447**
Lifetime abuse → poor emotion regulation	**.49**	**.11**		**.251**	**.722**
Executive function deficit→ poor emotion regulation	**.95**	**.24**		**1.43**	**4.67**
Lifetime abuse→ alcohol use severity	.03	.04		-.113	.062
Executive function deficit → alcohol use severity	.05	.08		-.232	.123
Poor emotion regulation → alcohol use severity	**.14**	**.03**		**.064**	**.223**
**Indirect Effect**					
Lifetime abuse → executive function deficit → poor emotion regulation → hazardous alcohol use	**.097**		**.031**	**.035**	**.158**
**Risky Sex Model 2**					
**Direct Effect**					
Lifetime abuse → executive function deficit	**.13**	**.05**		**.028**	**.248**
Lifetime abuse → sexual risk behaviors	.01	.05		-.002	.019
Executive function deficit → sexual risk behaviors	**.03**	**.02**		**-.048**	**-.005**
**Indirect Effect**					
Lifetime abuse → executive function deficit → sexual risk behaviors	**.078**		**.051**	**.002**	**.198**

### 4.4 Alcohol use

**Fig 1:**
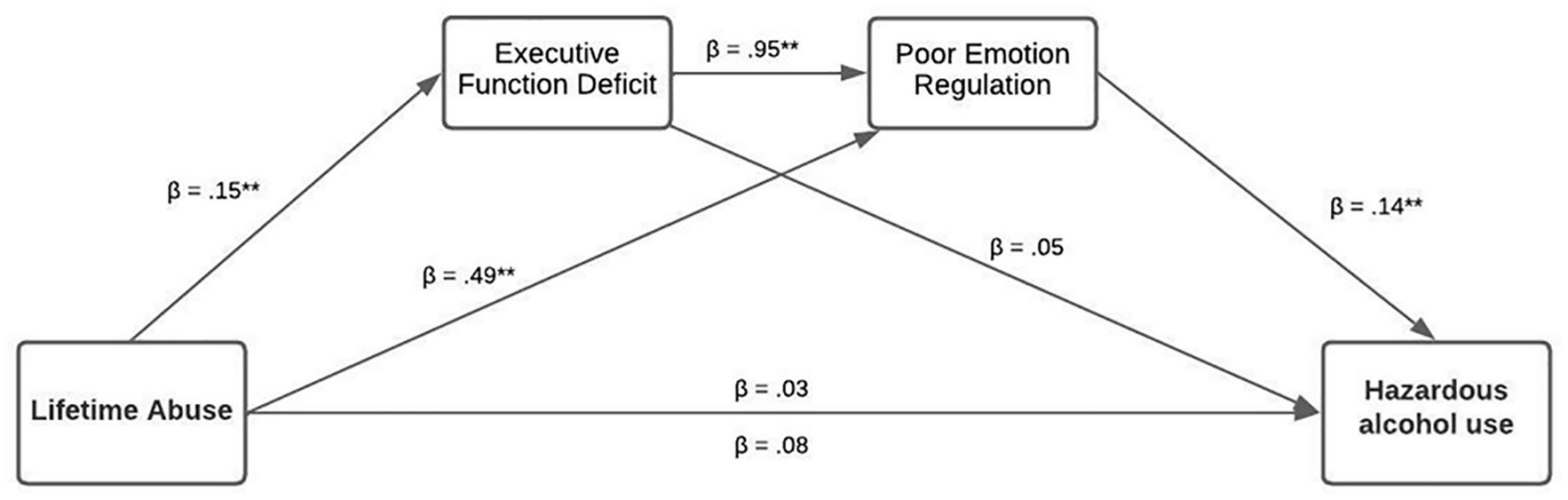
Mediation analysis between lifetime abuse, executive function deficit, poor emotion regulation, and alcohol use severity among women of color.

For Model 1, the total effect model was statistically significant; the mediators, executive function deficit, and poor emotion regulation were indirectly associated with lifetime abuse and hazardous alcohol use. See Fig 1, which illustrates the results of the mediation model analysis and Table 3 for the direct and indirect results. This was a fully mediated pathway. The indirect effect of executive function deficit via poor emotion regulation on the association between lifetime abuse and hazardous alcohol use was significant, with the total effect size accounting for 10% for a deficit in executive functioning and emotion regulation (indirect effect = .097, SE .031, 95% CI = .035 to .158; Fig 1).

### 4.5 Risky sex model

**Fig 2:**
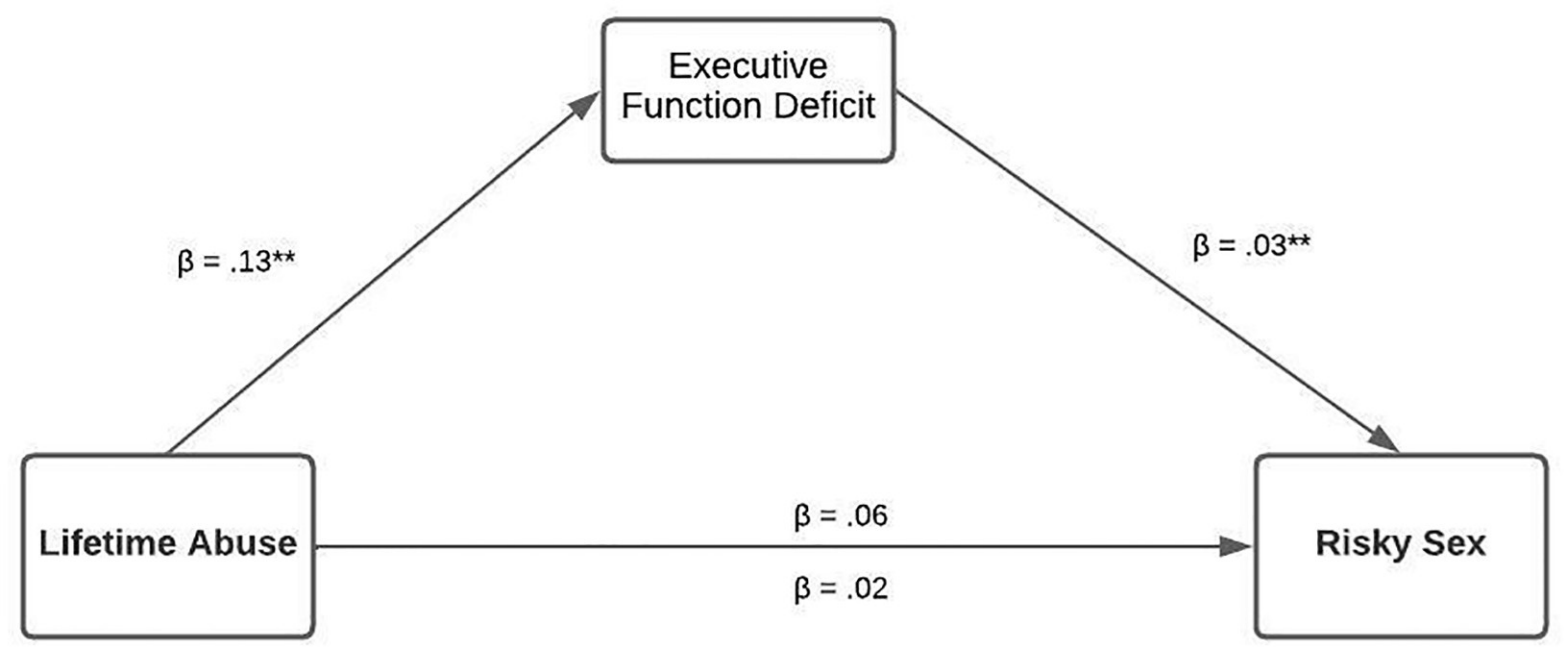
Mediation analysis between lifetime abuse, executive function deficit, and risky sex among women of color.

For the serial multiple mediation risky sex (Model 2), we measured the mediating effect of executive function deficit and poor emotion regulation between lifetime abuse and risky sex. The results of the model were partially significant. Only a deficit in executive function was indirectly associated with lifetime abuse and risky sex. See Fig 2, which illustrates the results of the mediation model analysis and Table 3 for the direct and indirect results. This was a fully mediated pathway. The indirect effect via deficit in executive function on the association between lifetime abuse and risky sex was significant, with the total effect size accounting for 8% (indirect effect = .078% SE = .051 95% CI = .002 to .198; Fig 2).

## 5. Discussion

This study has the potential to significantly add to the understanding of the mechanisms that contribute to increased alcohol use and risky sex in women of color with a history of lifetime abuse. To gain further insight we evaluated the mediating role of executive function and emotion regulation using a serial mediation model. First, we demonstrated that the pathway between lifetime abuse and increased alcohol use may occur through a cognitive-emotional mechanism which is consistent with the self-regulation theory. This theory proposes a higher-order cognitive process in connection with emotion regulation to produce an integrated behavioral response [39]. This model (i.e., executive function promoting emotion regulation) provides a framework for understanding how individuals interpret, respond, and adjust to negative experiences [27]. Developmental neuroscience suggests that adverse environments resulting from abuse and/or violence may expose children to chronic stress, which can disrupt brain architecture and impair the development of executive function, leading to cognitive problems in adulthood [40]. These types of environments can affect the organization and integration of neural circuits involved in emotion and cognitive functions, which can impair self-regulation and contribute to a host of maladaptive outcomes in adulthood [30, 31, 40, 41]. Our study is partially supported by a recent study demonstrating that exposure to childhood abuse was associated with a deficit in executive function and poor emotion regulation, which predicted future alcohol use behaviors later in life [42]. Although this study did not measure lifetime abuse, overwhelming research shows that adults who experienced childhood abuse have greater rates of intimate partner violence in adulthood [5, 6]. Results from this study are also similar to previous studies demonstrating greater alcohol use among women with a history of lifetime abuse [43–45].

In this study we showed that lifetime abuse may be associated with poor emotion regulation skills [41]. Adults who grew up in abusive environments may not have developed an intrinsic regulation mechanism because of the lack of support from their parents by been exposed to inconsistent or unpredictable parenting, which can disrupt the development of emotion regulation skills [30, 31]. Thus, their regulation of emotions denotes a rigid and maladaptive use of emotion regulation strategies and the inability to choose the most appropriate strategy for achieving goals [35]. This may lead to emotionally avoidant coping behaviors (e.g., alcohol use) which can be promoted by the executive function in women with a history of lifetime abuse.

Although we did not identify specific executive function processes or emotion regulation facets, this study demonstrated that the mechanism for hazardous alcohol use in women with lifetime abuse could be explained via the cognitive-emotional model. Research focusing on specific processes of executive function and emotion regulation may present valuable information on the mechanism for self-regulation among women with lifetime abuse.

We also tested the mediating role of executive function deficit and poor emotion regulation in the relationship between lifetime abuse and risky sex. We hypothesized that a deficit in executive function and poor emotion regulation would mediate the relationship between lifetime abuse and risky sex; however, our results showed that executive function deficit was the only significant mediator. Although more research needs to be done to understand the effect of emotion regulation on risky sex, the results showed that lower executive functioning is significantly associated with increased risky sex. Neuroscience research has shown that alterations in executive control in adults with a history of abuse have implications for decision-making and that these impairments may lead to a shift from flexible, goal-directed behavior to inflexible stimulus-response, making self-regulation more difficult [46, 47]. Another factor that may have the potential to influence risky sex is cognitive appraisals. For example, anticipated negative partner reaction has predicted women’s decreased sexual assertiveness and condom self-efficacy [48]. Our study was not designed to determine factors that may have contributed to this behavior. However, this is the first known study to analyze these relationships; thus, a longitudinal study focusing on specific executive function and emotion regulation processes for risky sex may be warranted.

### 5.1 Limitations and future directions

The results from this investigation should be interpreted in light of its several limitations. First, although we used validated self-report measures commonly used in the field, in-person interviews are subject to recall and social desirability bias. Second, we did not use specific processes to measure executive function and emotion regulation; instead, we used the cumulative assessment for these two variables. As mentioned earlier, disentangling the effects of executive function and emotion regulation systems will offer a better understanding of how these relationships may predict such outcomes. Third, since this was a cross-sectional design, we cannot make causal inferences or mediating effects. However, the independent variable of interest in the present study was collected retrospectively based on past exposure to abuse, while the mediators and outcomes were collected based on the present results. Therefore, the foundation for temporal ordering is present and adds support to the current design [37, 49]. Fourth, these variables may not be the only mediators in the models. It could be that there are other mediators which may not be in the model or even in the dataset. Nevertheless, our results in terms of the indirect effects suggest a theoretical contribution to the mechanism for cognitive-emotion regulation, which lays the foundation for a longitudinal mediation study focusing on the key study constructs. While we certainly acknowledge these various limitations, this work represents the early stages of a novel approach to understanding an understudied population. Finally, the sample size was small. Future studies with a larger sample size may be able to test whether emotion regulation mediates risky sex and confirm the cognitive-emotional model.

This is the first known study analyzing the indirect effects of executive function deficit and poor emotion regulation on lifetime abuse and risk behaviors, which provides a framework for understanding current work but also highlights how much remains to be clarified in future research. Although there is emergent research on the ability to regulate emotions, cognition, and behavior in response to internal and external demands in various populations, only one known prior study measured executive function as the pathway between early life adversity and performance [50]. It will be important to replicate our findings with a greater focus on the specific processes of executive function and emotion regulation related to self-regulation. It would also be important to include an interactive approach to the data analysis to clarify how different components of executive function and emotion regulation support the response to internal and/or external factors.

### 5.2 Conclusions

This study measured the mediating role of executive function and emotion regulation in the relationship between lifetime abuse and hazardous alcohol use and risky sex. Our findings suggest executive function and emotion regulation as a potential mechanism for hazardous alcohol use in women who experienced lifetime abuse (i.e., childhood abuse and intimate partner violence). Conversely, the indirect pathway for risky sex was only significant through executive function. We cannot determine the reason for this relationship, but we offered a few theories and proposed that a longitudinal study may provide further clarification. In sum, we showed that women exposed to lifetime abuse may have a deficit in their self-regulation mechanism through the cognitive-emotional pathway, thus relying on maladaptive strategies such as alcohol use and risky sex for regulating their negative emotions.
